# Hypermedia-based software architecture enables Test-Driven Development

**DOI:** 10.1093/jamiaopen/ooad089

**Published:** 2023-10-17

**Authors:** Andrew R Post, Nancy Ho, Erik Rasmussen, Ivan Post, Aika Cho, John Hofer, Arthur T Maness, Timothy Parnell, David A Nix

**Affiliations:** Huntsman Cancer Institute, University of Utah, Salt Lake City, UT 84112, United States; Department of Biomedical Informatics, University of Utah, Salt Lake City, UT 84112, United States; Huntsman Cancer Institute, University of Utah, Salt Lake City, UT 84112, United States; Huntsman Cancer Institute, University of Utah, Salt Lake City, UT 84112, United States; Huntsman Cancer Institute, University of Utah, Salt Lake City, UT 84112, United States; Huntsman Cancer Institute, University of Utah, Salt Lake City, UT 84112, United States; Huntsman Cancer Institute, University of Utah, Salt Lake City, UT 84112, United States; Huntsman Cancer Institute, University of Utah, Salt Lake City, UT 84112, United States; Huntsman Cancer Institute, University of Utah, Salt Lake City, UT 84112, United States; Huntsman Cancer Institute, University of Utah, Salt Lake City, UT 84112, United States

**Keywords:** high-throughput nucleotide sequencing, software, data management, cloud computing

## Abstract

**Objectives:**

Using agile software development practices, develop and evaluate an architecture and implementation for reliable and user-friendly self-service management of bioinformatic data stored in the cloud.

**Materials and methods:**

Comprehensive Oncology Research Environment (CORE) Browser is a new open-source web application for cancer researchers to manage sequencing data organized in a flexible format in Amazon Simple Storage Service (S3) buckets. It has a microservices- and hypermedia-based architecture, which we integrated with Test-Driven Development (TDD), the iterative writing of computable specifications for how software should work prior to development. Relying on repeating patterns found in hypermedia-based architectures, we hypothesized that hypermedia would permit developing test “templates” that can be parameterized and executed for each microservice, maximizing code coverage while minimizing effort.

**Results:**

After one-and-a-half years of development, the CORE Browser backend had 121 test templates and 875 custom tests that were parameterized and executed 3031 times, providing 78% code coverage.

**Discussion:**

Architecting to permit test reuse through a hypermedia approach was a key success factor for our testing efforts. CORE Browser’s application of hypermedia and TDD illustrates one way to integrate software engineering methods into data-intensive networked applications. Separating bioinformatic data management from analysis distinguishes this platform from others in bioinformatics and may provide stable data management while permitting analysis methods to advance more rapidly.

**Conclusion:**

Software engineering practices are underutilized in informatics. Similar informatics projects will more likely succeed through application of good architecture and automated testing. Our approach is broadly applicable to data management tools involving cloud data storage.

## Objectives

Bioinformatic data management in cancer research has migrated to the cloud.^1^ Reasons include availability of cloud-based analysis; storage and compute limitations at universities; federal support for cloud-based services;^2^^,^^3^ flexible cost models that minimize up-front investment; ability to distribute costs between institutions and investigators; and enabling Findable, Accessible, Interoperable, and Reusable (FAIR) data practices.^4^ FAIR data are needed to achieve precision oncology, the matching of treatments to a tumor’s molecular profile and patient characteristics.^5–8^ These benefits cannot be realized fully because tools for working with molecular data require high technological sophistication,^9^ especially in the cloud.^10^^,^^11^ Existing tools also have limited capabilities for core facilities to manage data on investigators’ behalf. Additionally, reliable distributed systems are challenging and costly to build,^12^ and conventional software engineering methods are too inflexible for the iterative and exploratory nature of bioinformatics.^13^ Usable, capable, flexible, and reliable software is needed to realize the full potential of cloud computing in cancer research.

Our long-term goal is to create, evaluate, and disseminate architectural patterns for informatics software for cloud data management. Our objectives in this project were to develop and evaluate a tool for managing bioinformatic data in the cloud, Comprehensive Oncology Research Environment (CORE) Browser. It aims to permit investigators and institutions greater control over data, decouple analysis from data management for maximum flexibility to choose analyses, and minimize investigator costs while achieving high reliability. Adoption of agile software development^14^^,^^15^ was our first step in addressing reliability, as we previously reported.^16^ Our next step is integrating automated testing^17^ into CORE Browser. Evidence suggests that automated testing leads to fewer defects, more frequent deployment, and more rapid feature development.^18^ However, software development teams must balance its benefits with the work required to develop tests.^18–20^ Architectural patterns gained through this effort could accelerate the informatics community’s work in supporting bioinformatic analysis and precision medicine.

CORE Browser development launched in mid-2021 to support our institution’s plans to store all high-throughput sequencing data in the cloud. We implemented a form of automated testing called Test-Driven Development (TDD)^17^^,^^18^ and evaluated the completeness with which we could test the code while minimizing the number of tests developed. This testing strategy is sufficiently mature after one-and-a-half years to present its strengths and limitations. Below we introduce CORE Browser’s architecture, show how the architecture and testing are synergistic, and evaluate how automated testing has performed.

## Background and significance

Our approach builds upon 3 developments. First, the software engineering community has created architectures supporting reliable interactions between locally hosted and cloud-based services, such as microservices.^21^^,^^22^ Second, there are mature information science methods for user-friendly and scalable access to complex heterogeneous data, such as hypermedia,^23^ the basis of the world wide web. Third, the software engineering community has advanced agile practices and automated testing to enable development of complex software.^18^ We believe that a new architecture informed by these advances can decouple data management from analysis, permitting a user-friendly data management system that changes relatively slowly while flexibly linking data to rapidly advancing analysis techniques. Without this, precision oncology may continue to be limited by the need for researchers to gain high technical fluency.

High-throughput sequencing data are often stored in Simple Storage Service (S3) buckets,^24^ originally part of Amazon Web Services (AWS)^25^ but now supported by major cloud service providers. AWS S3’s built-in tools include command line and complex web interfaces, and they require complex security settings,^26^^,^^27^ making them difficult for many researchers to use. Tools other than Amazon’s^28^^,^^29^ simplify access to S3 by implementing a “walled garden”^30^ with powerful methods for managing and analyzing data but limited flexibility, few features for core facilities to be data stewards, and limited support for investigators to analyze data with novel methods from third parties or their own lab. These systems may increase cloud costs and reduce flexibility by limiting direct access to underlying bucket storage; imposing higher compute and data egress charges than investigators could negotiate with cloud providers; limiting analyses to built-in methods; or requiring that data be exported out of the cloud with egress charges for analysis in other tools. More flexible architectures are needed while enabling ease of use.

Decoupling data management from analysis requires a service-oriented architecture (SOA),^18^ which organizes software as networked components. This approach makes software more difficult to reason about for developers than conventional monolithic architectures. Software built with SOA must be resilient to network glitches and third-party services being unavailable, among other concerns. While SOA^31^ is over two decades old and its microservices^21^ variant a decade old, developers still struggle with how to achieve reliable software with them. Automated testing may help manage complexity and ensure that changes to one service do not negatively impact others.

**Figure 1.**
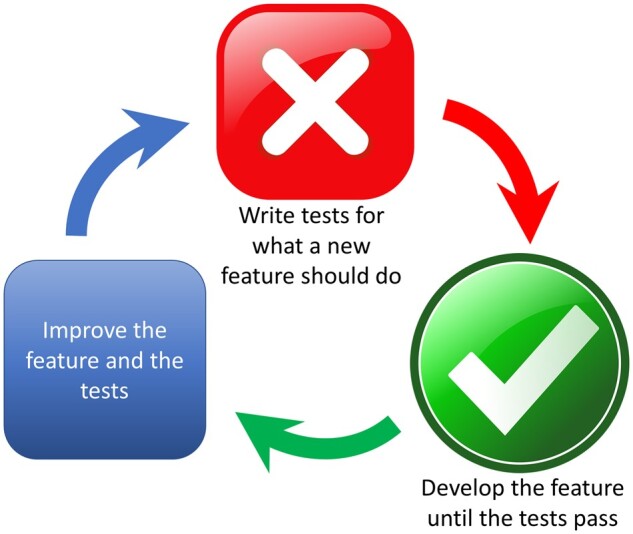
Illustration of Test-Driven Development, sometimes called red-green-refactor.

TDD has seen limited adoption but shows promise for increasing software reliability. In TDD, shown in Figure 1, tests are written before developing a new feature and serve as computable feature specifications. Tests fail initially (red), and pass as code is written that complies with the specifications (green). Refinement of the software, called refactoring,^32^ is performed with a goal of keeping all tests green. When a software defect is found, tests are written to reproduce the defect, which is considered fixed when the tests pass.

Evidence suggests that TDD improves code quality more than the usual practice of writing code first and developing tests later to verify that the code works.^18^ Reported benefits include higher code cohesiveness, looser coupling, fewer defects, fewer lines of code, and greater ease of making design changes.^33^^,^^34^ TDD may result in longer initial development time but possibly greater productivity during subsequent development phases due to higher code quality.^33^^,^^34^ TDD’s low adoption is typically blamed on unfamiliarity or low adoption of automated testing in general.^16^ In our experience, ambiguity about how a team should integrate it into their work is a barrier. TDD may be more likely to succeed when a software application’s architecture is designed for it,^18^ but what that means needs greater definition. The informatics community needs examples of successful TDD implementations.

We are developing CORE Browser using our new microservices- and hypermedia-based software development framework called Health Enterprise Analytics (HEA). Microservices are small software components implementing distinct units of functionality that are accessed over a network via Application Programming Interfaces (APIs) and are independently testable and deployable.^18^^,^^21^ Overall system behavior emerges from interactions between microservices. Hypermedia^23^ refers to a non-linear information medium on which the world-wide web is based. HEA aims to implement a cancer data-specific web in which users interact with data via hyperlinks to related data or actions that can be performed.^35^ We believe that HEA’s hypermedia architecture facilitates TDD due to the repeating pattern with which web clients and servers communicate with each other, links, which allow microservices to share an API design convention.

Contributions of this project are an evaluated architecture for self-service management of sequencing data in the cloud and an open-source implementation of our testing strategy. They are significant because self-service data access and sharing are believed to lower barriers to secondary use,^36–38^ and increased software quality may enable greater scientific reproducibility.^10^^,^^39–41^ Data sharing and reproducibility are central tenets of the FAIR guiding principles^4^^,^^5^^,^^42^ and National Institutes of Health (NIH) priorities.^43^

Additionally, informatics core facility effectiveness and investigators’ ability to build software would benefit from increased knowledge of software engineering practices. A recent NIH funding opportunity made enhancing research software reliability a priority.^44^ Furthermore, many academic informatics units have a service mission for their institution,^45^ and software engineering skills are critical for building deployable tools in clinical, research, and educational settings.

## Materials and methods

### CORE Browser

CORE Browser is developed by our institution’s Research Informatics Shared Resource. The project has 3 staff plus central architecture and database support. Personnel had no prior experience with TDD and limited experience with automated testing.

**Figure 2.**
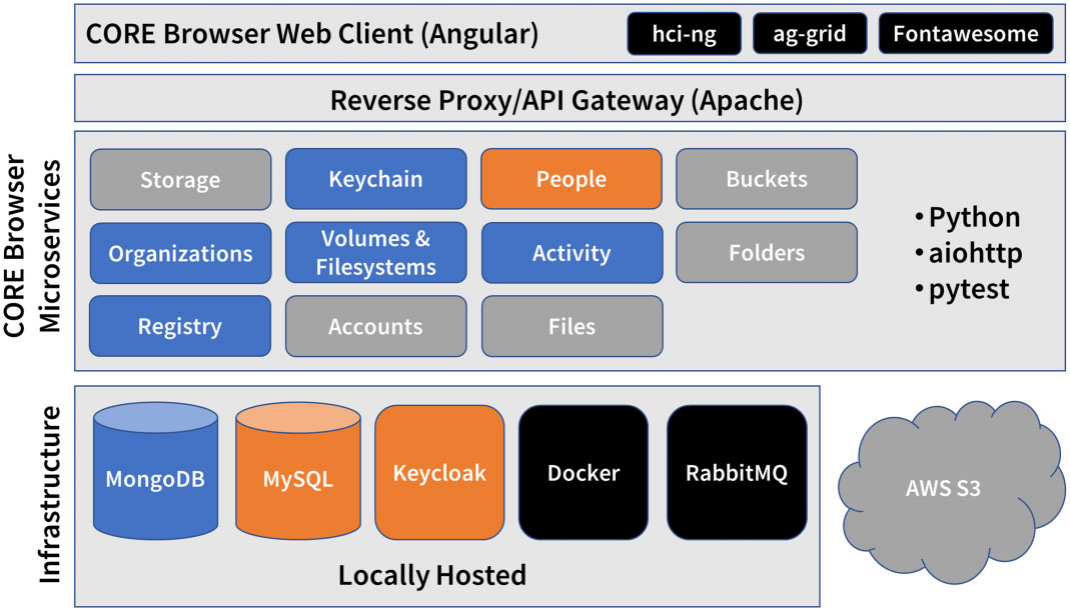
CORE Browser architecture stack diagram. CORE Browser implements a microservices architecture. Microservices are color-coded according to their data store: blue for MongoDB; orange for Keycloak, which stores user data in a MySQL database; and gray for AWS S3. They are deployed with Docker and exchange messages asynchronously using a RabbitMQ message queue, color-coded black to indicate that all microservices have them as dependencies. The web client code uses an internally developed Angular widget and style library, hci-ng, and the third-party ag-grid and Fontawesome libraries.

The architecture of CORE Browser is illustrated in Figure 2. Programming languages used include Python^46^ in the backend and Javascript/Angular^47^ in the frontend. Infrastructural components include MongoDB^48^ and MySQL^49^ databases; Keycloak,^50^ which provides OpenID Connect-based^51^ authentication and user management; Docker^52^ for automated deployment and establishment of a secure internal network for communication between components; RabbitMQ,^53^ a message queue for asynchronous communication between backend components; and AWS S3 buckets. There are currently 11 microservices, all implemented using the aiohttp web development framework^54^ with pytest^55^ for automated testing. An API gateway, implemented with Apache Server,^56^ provides OAuth2^57^-based authorization of API requests and serves as the sole access point to the internal network. The web client follows Single Page Application (SPA) design and makes asynchronous calls to the backend via the API gateway.

**Figure 3.**
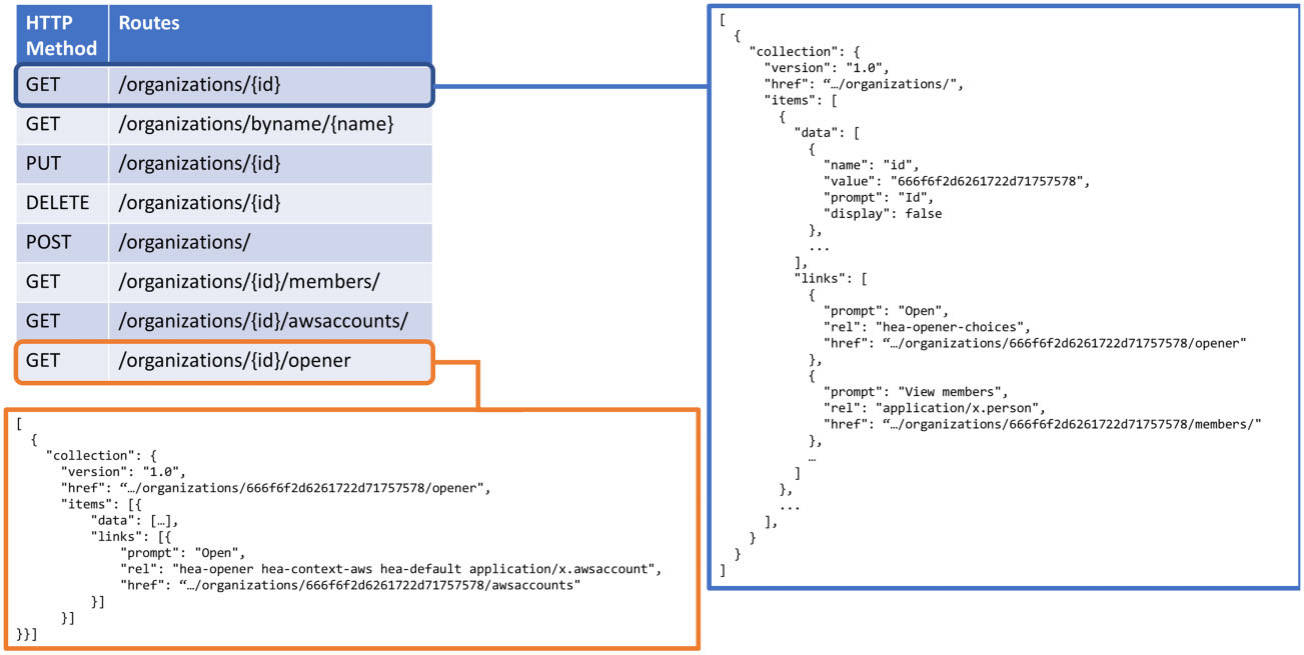
REST API convention in CORE Browser, illustrated with the Organizations microservice. The table, upper-left, shows the routes for getting (GET), updating (PUT), creating (POST), and deleting (DELETE) organizations. GET calls respond with JSON-formatted data, including an organization object, metadata for presenting it on screen, and links to actions that can be performed on the organization, upper-right. Links have a prompt property, one or more relation types (rel) that indicate the nature of the link, and the href of the link. Link hrefs are truncated in this figure to save space. The “opener” route responds with a link for getting the organization’s AWS accounts, lower-left.

The microservices, listed in Table 1, provide APIs for data stored in AWS and locally hosted services and databases. APIs adhere to a hypermedia-based and REpresentational State Transfer (REST)^35^ design, implementing at least 5 web service calls per endpoint following the CRUD (Create, Read, Update, and Delete) convention. Calls are illustrated in Figure 3 for the heaserver-organizations microservice, which manages information about research labs using CORE Browser. Requests and responses contain JavaScript Object Notation (JSON)-formatted bodies.^58^

**Table 1.** The locally developed backend components of CORE Browser.

Component	Description
heaobject	Software library containing data model classes.
heaserver	Software framework for building microservices.
heaserver-accounts	Microservice providing read-only AWS account information.
heaserver-activity	Microservice providing data on CORE Browser user activity.
heaserver-buckets	Microservice for read-write access to AWS S3 buckets.
heaserver-files-aws-s3	Microservice for read-write access to files stored in AWS S3.
heaserver-folders-aws-s3	Microservice providing an AWS S3 folder abstraction, since S3 buckets have no folders.
heaserver-keychain	Microservice for managing permanent and temporary user credentials.
heaserver-organizations	Microservice for read-write access to laboratory information (eg, membership, principal investigator, AWS account ids).
heaserver-people	Microservice for read-only access to user account information stored in Keycloak.
heaserver-registry	Microservice registry containing metadata such as access URL and data types served.
heaserver-storage	Microservice for querying the size of data stored in an S3 bucket stratified by storage class (eg, Standard, Glacier).
heaserver-volumes	Microservice for defining data storage locations. Volumes are equivalent to drives in Windows and volumes in MacOS, and each volume is associated with a filesystem object representing the kind of storage (eg, MongoDB, S3).

The web client and microservices implement a part of REST called Hypermedia As The Engine Of Application State (HATEOAS).^35^^,^^59^ The web client invokes an entry point URL to a network of information connected by hyperlinks. Subsequent requests from the web client are to URLs found inside link metadata that the server returned in response to previous requests. Links are annotated with relation types (“rel” attribute in Figure 3) to indicate the information that would be returned if the link were followed. Links may also be annotated with field labels and other user interface metadata for the web client. Depending on a link’s relation types, the web client may present links for the user to choose from or follow links automatically. Links and relation types are defined by the Internet Engineering Task Force (IETF) Request For Comments (RFC) 8288 web linking proposed standard.^60^ Multiple HATEOAS data and link transport formats are available. CORE Browser uses Collection+JSON^59^ (Figure 3), which additionally permits the server to send web form templates to the client to build forms dynamically. CORE Browser extends Collection+JSON with support for complex forms containing grids.

**Figure 4.**
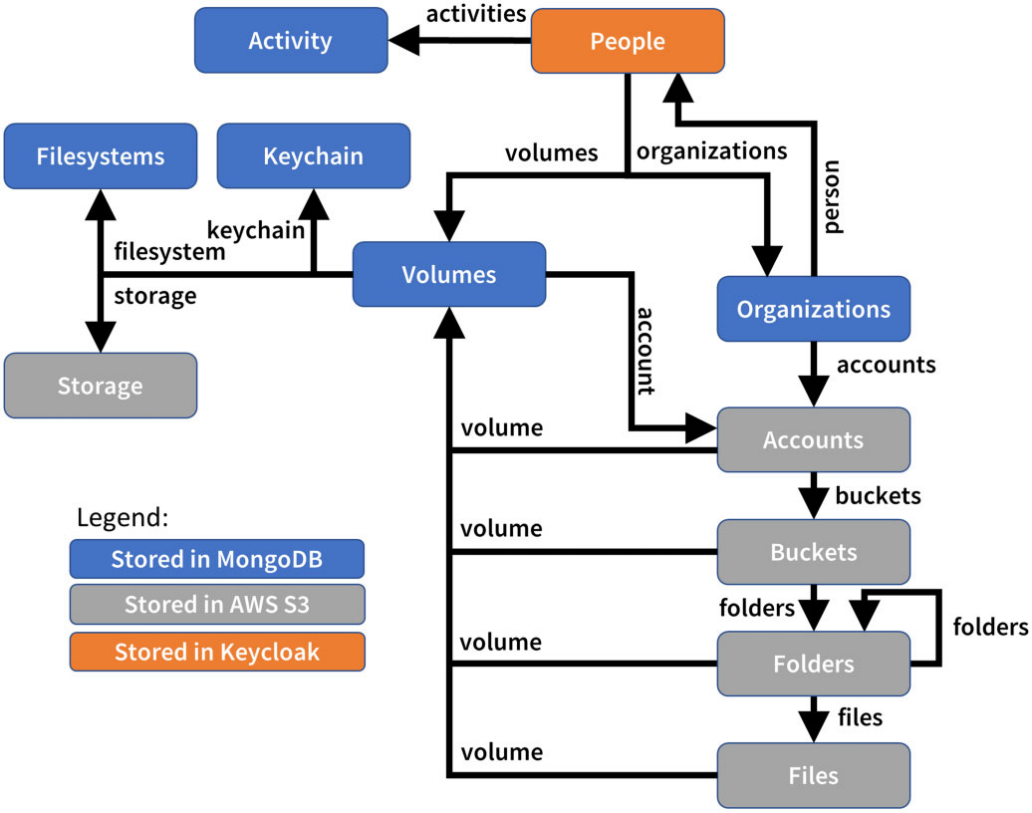
The network of links in CORE Browser. The orange, blue, and gray boxes represent the objects and data displayed in the application. The boxes are color-coded by where they are stored in the backend. The arrows represent links.

**Figure 5.**
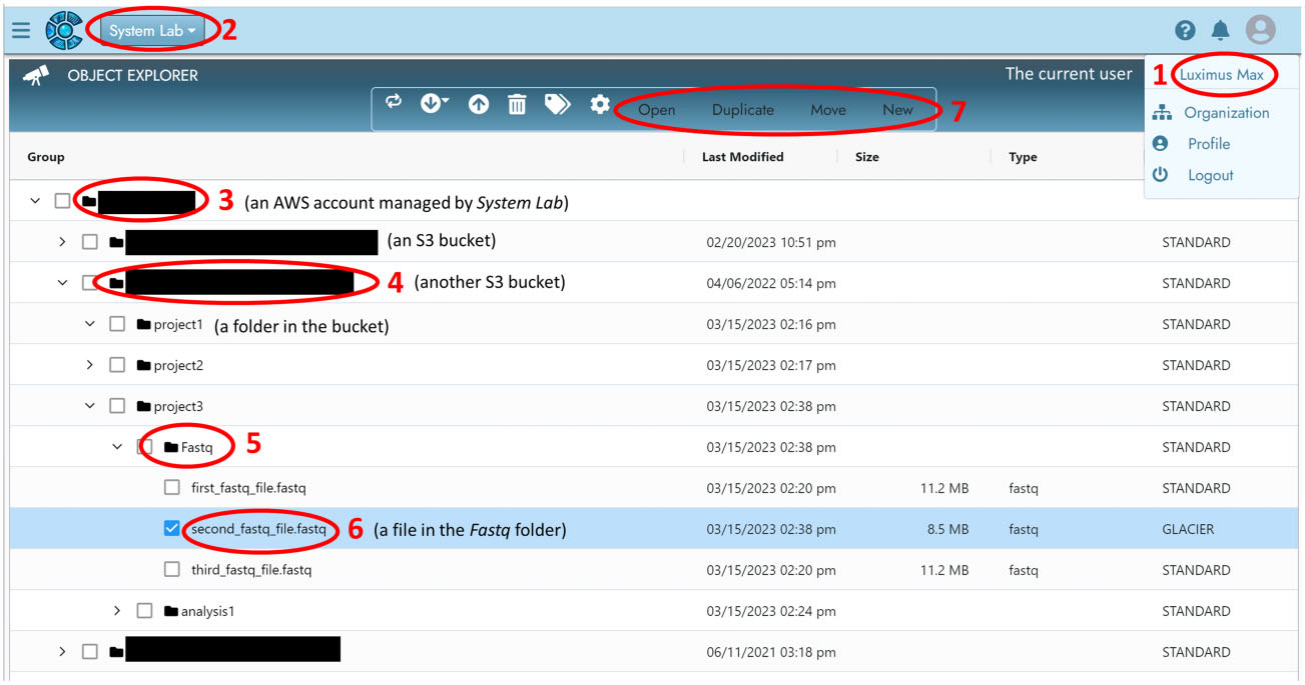
Screenshot of CORE Browser’s object explorer page, with annotations to illustrate how responses to the links in Figure 4 are rendered on screen. The user has logged into the application (#1), and they are authorized to access data from certain labs (organizations, #2). Upon selecting an organization in the dropdown, they are shown a tree of accounts (#3), from which they can open buckets (#4), folders (#5), and files (#6). Depending on the relation types in an object’s links, the web client might treat the object as a folder to be opened (accounts, buckets, and folders), or it might render the links as actions to be performed (#7, links to open, duplicate, move, or delete folders and files).

CORE Browser’s network of links is shown in Figure 4. The entry point is a URL for getting the current user. User objects have multiple links, such as to their lab membership (Organizations). From Organizations, a user might select a link to get the organization’s AWS accounts, and then the buckets, folders, and files the user can access. This data flow is realized in the application, shown in Figure 5, illustrating how links are automatically followed by the web client to render an organization list and a tree for selecting AWS accounts, buckets, and folders.

Major features include file and folder upload and download, copy, move, rename, permissions management, file metadata management, transfers to/from S3 archival storage, file version management for versioned buckets, and a “trash bin” for restoring and permanently deleting files from versioned buckets. CORE Browser simulates folders because S3 storage has limited folder support. The AWS accounts and buckets viewable by a user, and data they are permitted to edit, are controlled by their organization/lab membership and the AWS roles that are assigned to them. Lab managers and principal investigators can control lab membership, view and search lab members’ activity with their data, and view storage consumption.

### Testing approach

Because all messages between web client and server are formatted as Collection+JSON, and all microservices follow the same API pattern, we believed each microservice could parameterize and reuse a shared set of test “templates” with microservice-specific data and storage. In CORE Browser, these templates are methods of a set of mixins, a type of object-oriented class that augments the behavior of other classes via multiple inheritance.^61^ We implemented 121 test templates in 14 mixins to check the CRUD API calls of a single REST endpoint (Figure 3).

The parameterized test templates are “mixed” into at least 2 test suites per endpoint. One stores data in memory. The other stores data in a locally hosted S3 implementation, a MongoDB database running in Docker, or a dockerized Keycloak server, depending on the microservice. The S3 implementation is Moto, a software library that mocks many AWS cloud services.^62^ The former tests are designed to run in seconds, and the latter in minutes, making them suitable for unit and integration testing,^63^ respectively. Mixin tests that do not apply to a component are skipped automatically; for example, tests of APIs that modify data are skipped in read-only microservices. Most microservices implement a single REST endpoint and thus can execute up to 242 of these tests. The heaserver-volumes and heaserver-registry microservices each have 2 endpoints and could execute up to 484 mixin tests. Most microservices also implement a small number of custom tests.

CORE Browser library and framework components, heaobject and heaserver, implement testing differently. All heaobject tests are unit tests specific to that library. Heaserver has its own unit test suite for its distinct functionality, and it provides unit and integration testing of the mixin test classes.

### Descriptive analysis

We counted unit and integration tests executed for each backend CORE Browser component, including mixin tests. We also computed code coverage per component, defined as total statements executed during a test out of total statements present in the component’s source code directory.^19^^,^^20^^,^^64^^,^^65^ Code coverage is a widely used measure of testing completeness. The tests were executed 5 times on a Windows 10 Professional workstation with a 3.70 gigahertz 10-core Intel i9-10900K processor and 32 gigabytes of memory. We calculated code coverage with the open source pytest-cov library.^66^

## Results

CORE Browser is available as open source under the Apache 2 license, with the source code hosted on GitLab (https://gitlab.com/huntsman-cancer-institute/risr/hea). For deployment, Docker Compose manages service startup and shutdown. For integration testing, the open source testcontainers-python library^67^ performs this function instead.

TDD was achieved in CORE Browser because developing a new microservice begins by instantiating a code template using the Cookiecutter templating tool^68^ with the mixin tests pre-configured. Developers customize the code to create functionality and make the tests pass. Subsequently, developers customize the code with design and maintainability in mind, while ensuring that all tests continue to pass. The template was first created in July 2021, and all microservices created since then (9 out of 11 microservices) are based on it.

The CORE Browser backend has 13 989 source lines of code, not including tests. Heaserver provides 121 mixin test templates as described above, and backend components have a total of 875 additional tests. As shown in Table 2, 3031 tests were executed across all backend components per trial. Overall backend code coverage was 78%, with coverage per component ranging from 53% to 93%. All tests passed. Execution time was an average of 46.46 min (95% CI, 44.84-48.08; 5 trials). Nearly all the time was spent executing the integration tests. The unit tests for each component took a few seconds to run.

**Table 2.** Test counts and coverage by CORE Browser backend component.

Component	Storage	# unit tests	# integration tests	# total tests	Coverage (%)
heaobject	N/A	517	0	517	83
heaserver	N/A	451	210	661	79
heaserver-accounts	S3	13	10	23	53
heaserver-activity	MongoDB	121	121	242	90
heaserver-buckets	S3	62	66	128	69
heaserver-files-aws-s3	S3	22	25	47	62
heaserver-folders-aws-s3	S3	78	89	167	65
heaserver-keychain	MongoDB	121	121	242	93
heaserver-organizations	MongoDB	106	105	211	81
heaserver-people	Keycloak (MySQL)	18	6	24	70
heaserver-registry	MongoDB	123	124	247	89
heaserver-storage	S3	4	4	8	86
heaserver-volumes	MongoDB	257	257	514	90
Total		1893	1138	3031	78

## Discussion

CORE Browser complements our home-grown high-throughput sequencing laboratory information management system (LIMS) called GNomEx.^69^ The GNomEx software supports high-throughput sequencing facility workflows and has limited support for investigators to manage data on local storage and share data with collaborators. Other LIMS’^70–73^ similarly focus on sequencing workflow, or they store data in custom databases rather than flexible cloud storage that we believe will “future-proof” data storage to the greatest extent. CORE Browser provides data management and sharing that is unavailable in LIMS software and supports investigators with self-service data access. Its architecture permits extension to other cloud providers and our institution’s high-performance computing facility. Sharing data with the public is likely better served by other efforts such as dbGap.^74^

TDD is compatible with microservice-based web applications like CORE Browser.^75^^,^^76^ We achieved TDD by developing a small number of test templates in framework software (heaserver) that are parameterized and executed by each microservice, relying on CRUD and HATEOAS design conventions. Test failures indicate an inadvertent departure from our API convention. Implementing reusable tests was critical to ensuring the broadest code coverage. A tradeoff is that the microservices have a common dependency that could limit their independent testability. Microservice architectures frequently have shared components to reduce duplication, and in practice changes to heaserver have rarely changed a microservice’s API. CORE Browser’s microservices frequently depend on different heaserver versions and still function as a complete system.

All forms of automated testing can increase the confidence with which developers can change code without breaking it.^77^ Testing after development was described above. Others include acceptance testing, where tests formally describe user-oriented software requirements, behavior driven development, in which expected software behavior is expressed using natural language constructs,^78^ and end-to-end testing, which tests whole user workflows. All may be employed in the same application. Implementing the testing described in Methods with something other than TDD would likely be achievable but at higher cost because TDD is synergistic with heaserver’s design. However, adopters of our approach could employ other forms of testing elsewhere in their code.

Ensuring that all tests run in at most 1 h is a best practice.^18^ Tests are then compatible with an advanced agile practice called continuous integration and delivery,^18^^,^^33^ an automated process of releasing a new software version after every change made by a developer, ideally daily. All tests are executed upon every change that is committed to version control,^79^ and a change is rejected if tests fail. If defects are discovered, developers add tests to the system to replicate and support fixing the defect. While CORE Browser’s overall test execution time is under an hour, we only run tests on microservices that have changed. Tests of any individual microservice take at most 15 min, thus we have room to add tests and increase code coverage without exceeding the 1-h threshold. Implementing continuous integration and delivery is a future goal of the project.

While code coverage is a widely used measure of testing completeness, what constitutes good coverage remains a matter of debate. Google, for example, reported a practice in which 60% coverage is “acceptable,” 75% is “commendable,” and 90% is “exemplary.”^19^^,^^20^ Other sources refer to an informal 80% standard.^64^^,^^80^ CORE Browser’s backend code coverage approaches 80% and is commendable by Google’s standard.^20^ All backend components meet or exceed Google’s acceptable standard except heaserver-accounts due to limitations in Moto that prevent complete testing of its features.^62^ Lack of tooling is a relatively common reason cited for lack of TDD adoption and can result in lower test coverage.^33^

While coverage percentage can be a useful metric, it is probably not the most important insight to gain from measuring code coverage. The Pareto Principle^81^ suggests that 20% of code contains 80% of defects. This code may be enriched in complicated processes that are more likely to contain defects, more difficult to test completely, and therefore less likely to be fully tested. In CORE Browser, the AWS-related services have lower test coverage than those that use MongoDB as a data store due to challenges with Moto described above. Limited ability to test interactions with cloud services is likely an emerging problem for informatics and other data intensive application areas. Code coverage reveals this and tells us which components to target for testing enhancements. With many code coverage tools, coverage gaps can be identified down to the statement.

In addition to lower test coverage of AWS-related microservices, we have not automated front-end testing due to its labor-intensiveness even with tooling like Selenium.^82^ Instead, we manually test major front-end features and the user actions described above. We believe front-end testing is primarily valuable as part of end-to-end testing. Weighing a manual approach versus automation is a common engineering tradeoff, and comprehensive automated testing does not fully replace the need to test manually to identify unexpected user actions.^77^ However, as CORE Browser grows, these tradeoffs will likely start favoring addition of automated front-end testing in the future.

Good test coverage with all tests passing is an indirect measure of software quality and reliability, even if it may be the best that can be done before software is released. CORE Browser’s anticipated release is summer 2023. After it has been in users’ hands for a while, more direct measures of quality, architecture, and testing strategy will be possible. These include measures of stability such as change failure rate (how often a change introduces a defect) and recovery failure time (how long to recover from failures). Measures of throughput include lead time (how long it takes for a 1-line change to go from idea to working software) and deployment frequency (how often changes are deployed into production).^18^ Continuous integration is a prerequisite to these measurements. Achieving good change failure rate, recovery failure time, lead time, and deployment frequency are long-term goals of the project.

Prior to this project, we had made little use of automated testing, nor were there similar concurrent projects employing other testing approaches, precluding systematic comparison. As with prior studies, we would expect to find increased quality and decreased productivity, possibly resulting in higher productivity after initial implementation due to fewer defects. One study in an academic setting found increased productivity resulting from TDD,^83^ but the reason why is unclear.

The informatics literature has few publications like ours about software engineering challenges.^16^ While insufficient research funding for software development is a plausible explanation, evidence also suggests lack of awareness of best practices among many laboratories that build software.^16^^,^^41^^,^^44^ More work is needed to increase the reliability of applications arising from academic informatics units, enabling greater contributions to their institutions’ operations.^45^

User-friendly management of high-throughput sequencing data stored in S3 has been the primary aim of CORE Browser’s development, and as such the project appears to be unique. Amazon’s tooling, described above, is designed for technology savvy individuals and spread across powerful but complicated services. Third-party applications for working with genomic data in the cloud are generally investigator rather than core facility focused, or they may primarily implement analyses, which we believe are more flexibly provided as separate software. Example analysis tools include Seven Bridges,^28^ Cancer Genomics Cloud, and RAPTOR.^84^ Increasingly researchers store data in their own S3 buckets and access them through analysis tools, making separate data management tools like CORE Browser feasible.

Other tools such as Filesystem in Userspace (FUSE)^85^ allow “mounting” S3 storage as a drive on the user’s personal computer but lack tools for cores to manage data, and they provide limited support for large files and S3-specific functionality. Globus^86^ implements transfer of large files from one desktop computer or server to another, including S3 buckets, rather than management of data within AWS. Galaxy^9^ provides user-friendly data analysis and has similar user-facing goals to CORE Browser except in analysis. Cyberduck^87^ is a storage browser for online services including S3, but it has neither genomic nor scientific features. Separating bioinformatic data management from analysis appears to be novel, as does CORE Browser’s application of hypermedia to facilitate automated testing. We plan to carry out usability testing and investigate linking scientific analysis platforms to our software in the future.

CORE Browser is planned as the user-facing gateway to our cancer institute’s comprehensive linked databases. Beyond genomic data management, these include specimen tracking, clinical, and population research data. CORE will comprise a suite of software components, connected through a data hub, that encompass highly integrated cancer research data capture, browsing, query, visualization, and mining. We plan to implement automated testing across all parts of CORE over time.

## Conclusion

Software engineering practices are underutilized in informatics. After one-and-a-half years of CORE Browser development, we conclude that similar future informatics projects will be more likely to succeed with consistent application of good architectural practices and automated testing. Architecting to permit test reuse through a hypermedia approach was a key success factor for our testing efforts. Separating bioinformatic data management tools from analysis may have substantial flexibility benefits.

## Data Availability

The data underlying this article are available in the Dryad Digital Repository, at https://dx.doi.org/10.5061/dryad.pvmcvdnrv.

## Notes

**Author Contributions:** A.R. Post is considered co-senior author of this work.
